# Multimodal approach to rehabilitation of the patients with lateral epicondylosis: a case series

**DOI:** 10.1186/s40064-016-3375-y

**Published:** 2016-10-05

**Authors:** Alexandre Marcio Marcolino, Lais Mara Siqueira das Neves, Bruna Gabriela Oliveira, Aline Aguiar Alexandre, Guilherme Corsatto, Rafael Inacio Barbosa, Marisa de Cássia Registro Fonseca

**Affiliations:** 1 Laboratory of Assesment and Rehabilitation of the Locomotor Apparatus (LARAL), Federal University of Santa Catarina, Campus Araranguá, Rua Pedro João Pereira, 150, Florianópolis, SC CEP 88905-120 Brazil; 2 Rehabilitation and Functional Performance, University of São Paulo, São Paulo, SP Brazil; 3 Municipal Institute of Higher Education Catanduva, São Paulo, Brazil

**Keywords:** Rehabilitation, Musculoskeletal manipulations, Tennis elbow, Evaluation

## Abstract

**Purpose:**

The objective of this study was to evaluate the effectiveness of mobilization with movement and kinesiotherapy in the treatment of patients with lateral epicondylosis.

**Methods:**

This cases series included eight volunteers who had chronic lateral epicondylosis. The patients were treated with stretching, massage deep transverse at the lateral epicondyle and mobilization with movement associated with eccentric exercise. The mobilization with movement that consisted of a force of lateral glide of the proximal forearm. We performed twelve sessions, twice a week for 45 min/session. All patients underwent an evaluation with a visual analog scale and functional assessment through questionnaires patient-rated tennis elbow evaluation (PRTEE) and disabilities of the arm, shoulder and hand (DASH), before and after the treatment. Data were analyzed by student’s *t* test (p < 0.05).

**Results:**

The results showed statistical differences in pain symptoms before and after treatment, in the analysis and functional assessment through both questionnaires comparing the pre and post treatment.

**Conclusion:**

The data obtained in this study demonstrates improvement of the function and pain status of the sample investigated.

## Background

The lateral epicondylitis (LE) of the elbow pathology caused by injury of musculotendinous in the extensor carpi radialis brevis muscles, and can also affect the extensor digitorum communis (Garg et al. 2010; Nirschl and Ashman 2003; Kroslav and Murrel 2007) and in some cases affects the extensor carpi radialis longus (Freitas 2005), all in the level of their common origin at the lateral epicondyle. It is characterized by pain at the lateral epicondyle exacerbated with wrist extension, supination and grip strength (Garg et al. 2010; Struijs et al. 2005) and sensitivity in muscle mass proximal (Trudel et al. 2004).

Despite of lateral epicondylitis is also referred to as “tennis elbow”, is present in less than 5–10 % of players (Garg et al. 2010) and can appear in various tasks of excessive and repetitive effort involving movements gripping or pronation–supination (Garg et al. 2010; Kroslav and Murrel 2007; Anderson and Rutt 1992) as carpenters, surgeons, musicians, housewives (Anderson and Rutt 1992), occupations in construction, installation, manufacturing and feeding process (Trudel et al. 2004). There is a probability of 40–50 % of tennis players occur describe an annual incidence of 1–3 % in general population (Garg et al. 2010; Bauer and Murray 1999; Meyer et al. 2002).

Thus, the principle behind many treatments is to reduce the force acting at the origin of the extensor muscles of the wrist allowing time for recovery to occur (Kroslav and Murrel 2007). Nirschl (2003) treatment divided into five phases: (1) pain relief with controlled hemorrhagic exudation; (2) promote the healing of tissue specific; (3) promote general fitness; (4) control of workload forces; (5) removal of the pathological tissue by surgery. The first four stages are similar to those found in Freitas (2005). Can be seen, then, that the ultimate goal is not only pain relief but also promote normal muscle strength and conditioning for performance of activities.

For conservative treatment of lateral epicondylitis uses several resources, pharmacological (Frizziero et al. 2016) and non-pharmacological, such as: physiotherapy modalities, strengthening and stretching exercises, laser therapy, massage, electrotherapy, manual therapy (Trudel et al. 2004; Struijs et al. 2004), ionization with diclofenac (Trudel et al. 2004), injections of corticosteroids and orthotic devices (Borkholder et al. 2004; Struijs et al. 2004), concept of mobilization with movement (Vicenzino et al. 2007) and neural slide (MacDermid et al. 2010).

Trudel et al. (2004) and Bisset et al. (2005) performed two systematic reviews to investigate the effectiveness of conservative treatment in LE. The authors reported beneficial effects with the use of therapeutic ultrasound, phonophoresis, with or without friction massage and exercises, the ionizing currents, pulsed electromagnetic fields of low intensity laser, acupuncture, mobilizations and manipulations that led relief the pain status of patients assessed in several studies. In the study by Trudel et al. (2004), the authors also highlighted the positive effects such as improved pain, grip strength and hand function. Bisset et al. (2005) concern about the interaction of intervention techniques in this pathology. The authors conclude that there are a number of studies in various therapeutic interventions for LE and demonstrated the various conservative treatment options for the management of lateral epicondylitis and also the need for further research on the topic.

Cullnane et al. (2014) made a literature review to evaluate the effectiveness of eccentric exercise in patients with lateral epicondylitis, the authors observed in the evaluated studies improved function, pain and grip strength.

According Mulligan (1993), mobilization with movement is a form of manual therapy, based on sustained sliding sideways at the elbow joint associated with concomitant physiological movement. Miller (2000) reports the use of mobilization with movement for lateral epicondylitis as the primary modality for the correction of “positional fault of the elbow joint complex mimicking a pathological contractile element of the common extensor beam”. Pagorek (2009) reports in their literature review that the technique of mobilization with movement could be further studied to explore the effects caused in individuals with lateral epicondylosis and it would be important to have a long-term evaluation of these patients to observe a beneficial effect of this technique.

According to the literature found and described who underwent an intervention in patients with lateral epicondylosis mostly describe need for further research to evaluate the different techniques and therapeutic resources that were part of the treatment of patients with this condition, this way, we prepared this cases series, with the aim of observing the effectiveness of mobilization with movement association with exercise eccentric, massage and stretching in the rehabilitation of patients with lateral epicondylosis.

## Methods

The study included eight volunteers with tendinopathy at the lateral portion of the elbow (lateral epicondylosis), in total nine elbows, volunteers were sent by public health system of the city of Catanduva-São Paulo, Brazil.

### Criteria for inclusion and exclusion

Were included in the study subjects above 18 years with complaints of pain in the unilateral or bilateral lateral epicondyle and along with the achievement of specific tests positive for lateral epicondylosis (Cozen test, Mill’s test and Handshake test (4) and minimum time of 2 months of the pathological frame. The study excluded individuals with any type of fracture in the elbow, wrist and fingers region who undertake the range of motion of these joints.

### Study design

The eight volunteers who participated in this study were treated with mobilization with movement, massage, stretching and eccentric strengthening of the wrist extensor muscles of the affected side: 12 sessions, two times per week with an average duration of 45 min per session were performed.

#### Procedures performed with the volunteers

Passive stretching exercises in cervical spine and upper limb—stretching kept for 1 min each.

**Fig. 1:**
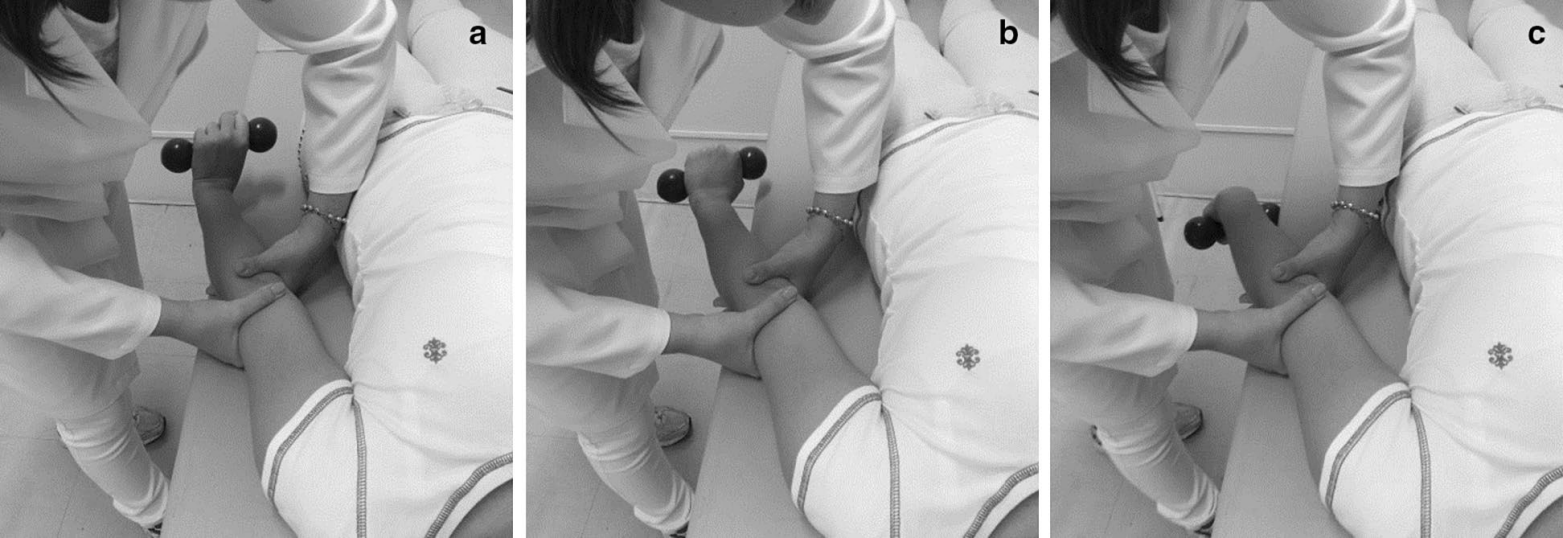
**a** Early mobilization with movement with the wrist in extension; **b** further mobilization with movement with the wrist in neutral position; **c** completion of mobilization with movement with the wrist in flexion

Muscle strengthening—three sets of ten reps. Eccentric exercise of the extensor muscles with the aid of load 1 kgf, associated with mobilization with movement (Fig. 1).

Transverse massage in the region of origin of the tendons of the extensor muscles of the wrist and fingers for 2 min and inhibition of points of tension in the belly of the extensor region.

Patients underwent an initial evaluation with a functional assessment of upper limb through the questionnaire disabilities of the arm, shoulder, and hand (DASH) and specific questionnaire for lateral epicondylosis, Patient-rated tennis elbow evaluation (PRTEE) and assessment of pain with a visual analog scale. After the end of the 12 sessions, the patients were reassessed following the same procedures of the initial assessment, and at the end of the completion of the revaluation was made available to patients a booklet of exercises in order to extend the possible beneficial results achieved through treatment.

### Patient-rated tennis elbow evaluation (PRTEE)

The questionnaire assesses the degree of difficulty that the volunteer had the previous week compared to their affected upper limb. The score ranges on a scale of 0–10, a zero means that the individual presents no pain and ten means it has the worst pain imaginable. The functional assessment of upper limb affected by lateral epicondylosis occurs through the specific activities carried out by the voluntary and habitual and its difficulty or not to perform (Andrade et al. 2001).

### Disabilities of the arm, shoulder, and hand (DASH)

The functional evaluation of the upper limbs was through the DASH questionnaire contained 30 questions capable of revealing the degree of dysfunction and symptoms, in which, the lower the score, the better upper limb function (Orfale et al. 2005; Camargo 2007).

### Ethical approval

This study was approved by the research ethics committee of the University of Medicine of Catanduva with case number: 298 762. All volunteers who agreed to participate read and signed a term informed consent.

### Data analysis

Data for visual analog scale, the DASH and PRTEE were analyzed by student t-test statistical method through Biostate 5.0 software.

## Results

Results related to the eight volunteers, data obtained through an evaluation before the treatment period (Table 1), age, body mass index (BMI), gender, dominance and affected side.

**Table 1:** Demographic data related to the eight volunteers, mean and standard deviation (SD)

Demographic data		
Age	41.87	±14.87 SD
BMI	23.88	±3.13 SD
Gender	Female	Male
	6	2
Dominance	Right-handed	Sinister
	8	0

	Left	Right	Bilateral
Affected side	3	4	1

Data obtained using a visual analog scale (VAS) showed a large reduction in pain after treatment period in all patients (Fig. 4)

**Fig. 2:**
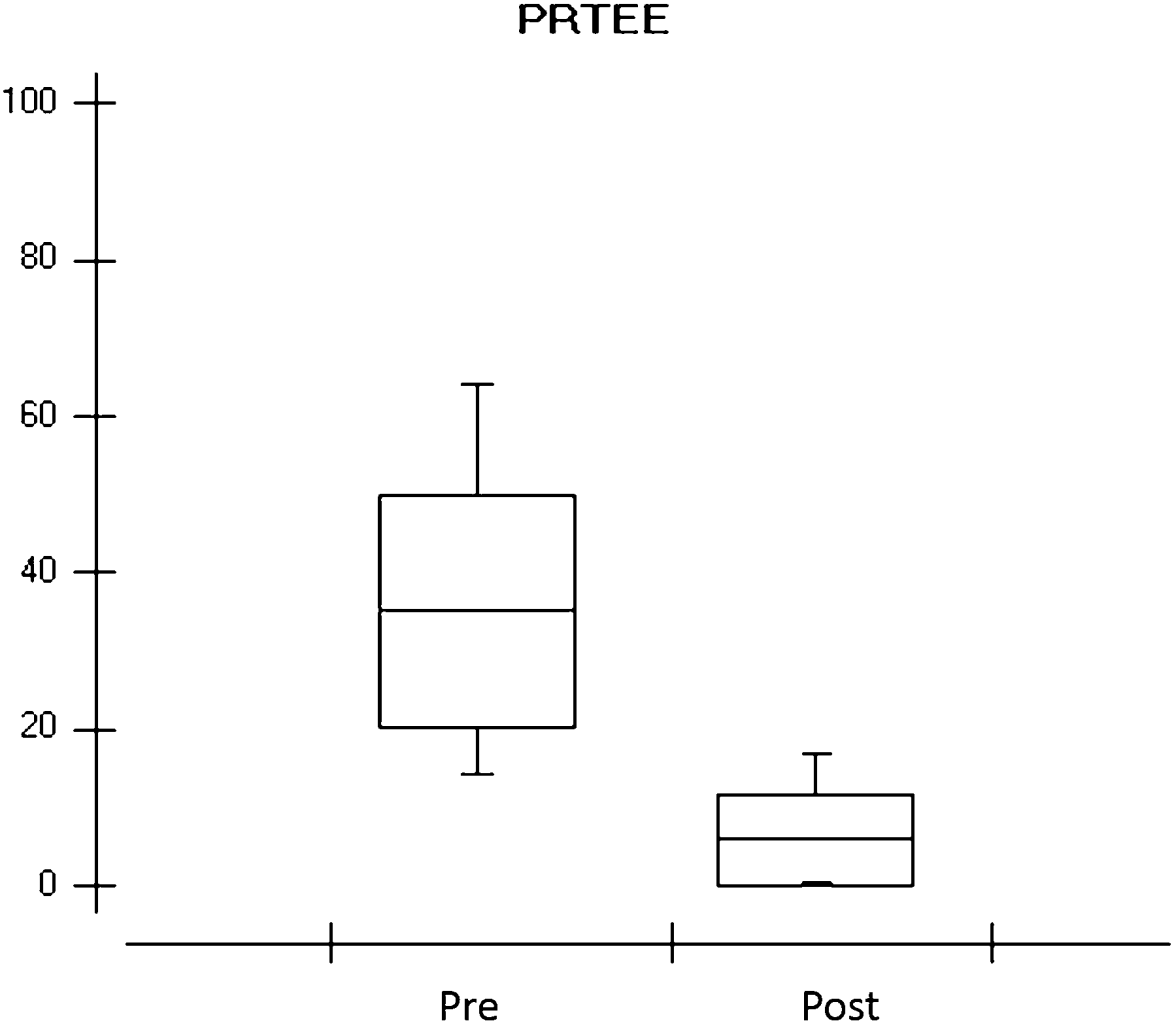
*Graph* showing the response of the PRTEE questionnaire of the eight volunteers respectively in the pre and post treatment

The response obtained in the PRTEE questionnaire (patient-rated tennis elbow evaluation) showed great improvement after the treatment period in the evaluation of the volunteers (Fig. 2).

**Fig. 3:**
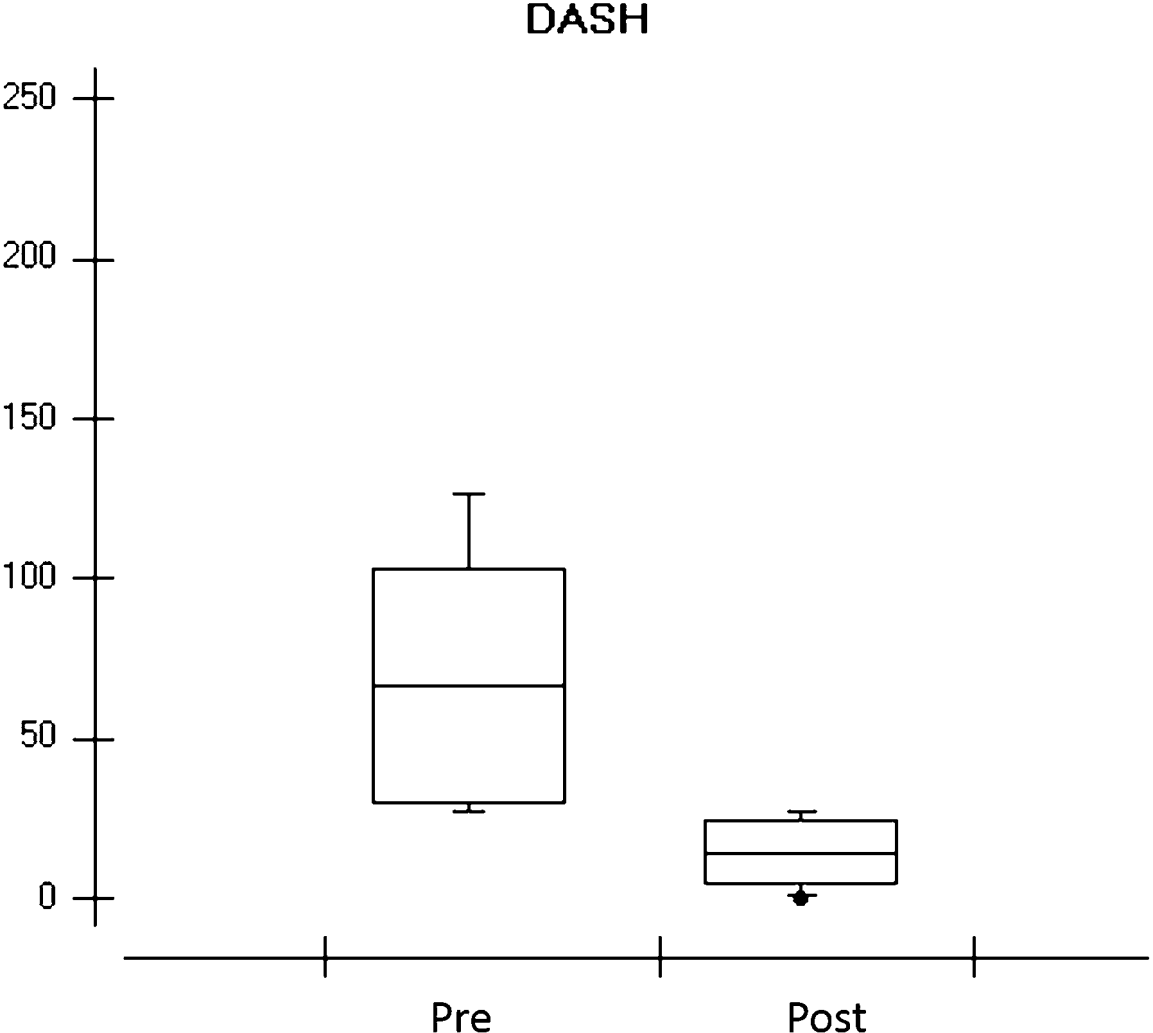
*Graph* showing the response of the DASH questionnaire eight volunteers respectively in the pre and post treatment

The data acquired through the DASH questionnaire (disabilities of the arm, shoulder, and hand) showed a noticeable difference when comparing the pre and post treatment (Fig. 3).

**Fig. 4:**
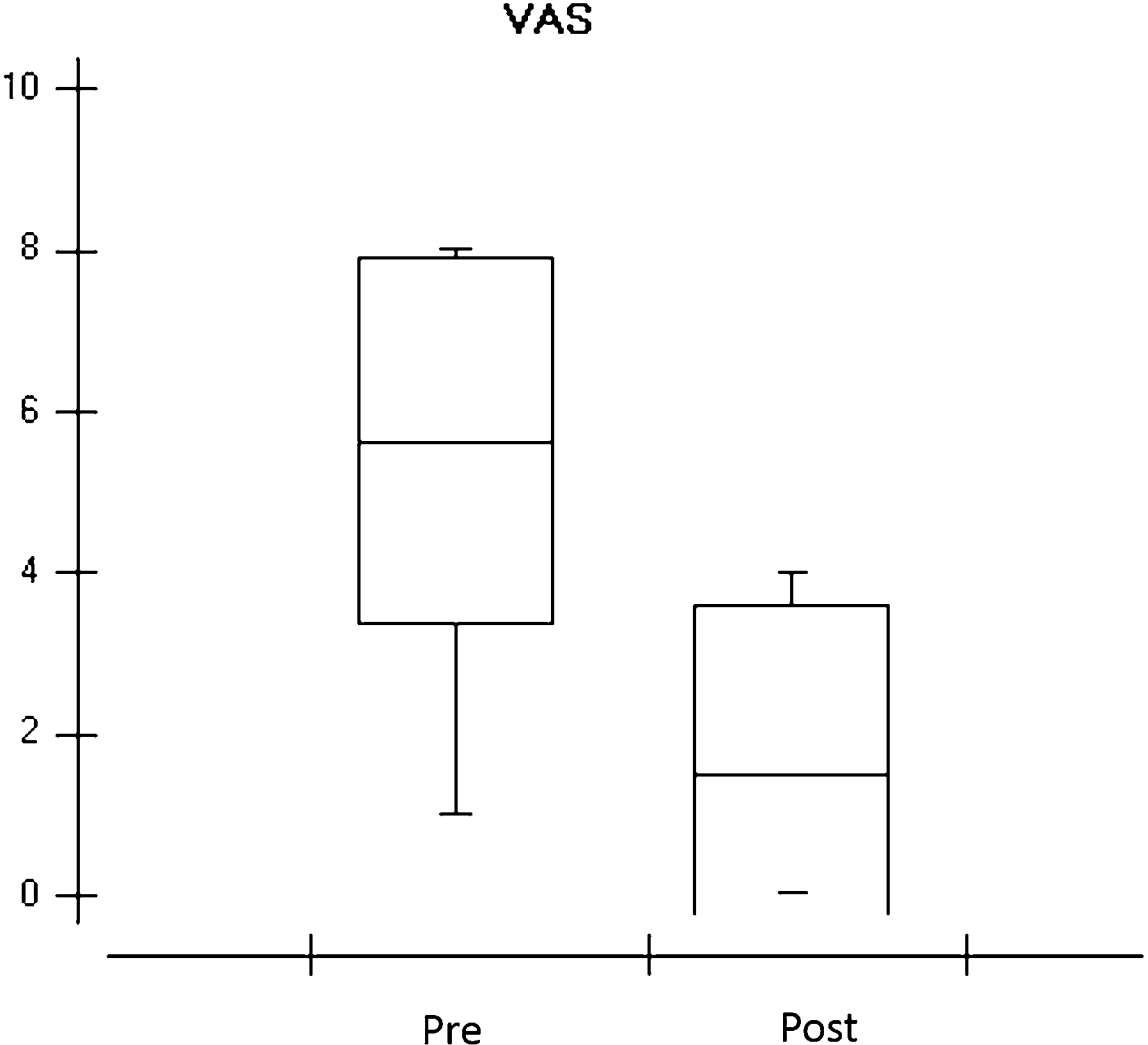
Graph on the visual analogue scale showing the pain reported by eight volunteers in the pre and post treatment

The comparison of the data pre and post treatment was statistical difference (p < 0.01) in relation to pain and upper limb function (Table 2).

**Table 2:** p value refers to comparison of data on pre and post treatment with the *VAS* visual analogue scale, *DASH* disabilities of the arm, shoulder, and hand questionnaire and *PRTEE* patient-rated tennis elbow evaluation questionnaire, for all volunteers

	p value		
	VAS	PRTEE	DASH
Pre X post treatment	0.01	0.01	0.01

## Discussion

The aim of our study was to verify the efficiency of the multimodal treatment with mobilization with movement associated with eccentric strengthening, transverse massage and stretching in the treatment of eight volunteers with symptoms of lateral epicondylosis.

According to Abbott and collaborators (2001), the mobilization with movement is a manual therapy strategy that uses forces that influence in the biomechanical joint alignment which can facilitate the desired movement.

In our study, the data obtained at the end of treatment using the visual analogue scale (VAS), disabilities of the arm, shoulder, and hand (DASH) and patient-rated tennis elbow evaluation (PRTEE) were effective and embodying the idea that mobilization with movement is an effective technique when used in the treatment of lateral epicondylosis.

In this study, pain was assessed using VAS, an improve was noted in pain status of the volunteers comparing before and after 12 sessions of treatment (p < 0.01). Only the fifth volunteer did not observe difference in pain relief, since the seventh volunteer complained little pain at the beginning of treatment and after the treatment, the pain was cured, and when the other volunteers observed, there was overall improvement in pain symptoms.

Regarding PRTEE and DASH questionnaires obtained, both had a decrease in post-treatment period with a statistical difference when comparing the pre and post treatment with p value <0.01.

We must consider that we did not get an even better result, because the majority of patients during the treatment period did not stop their occupational activities that may be the offending agent of this injury. For this study, volunteers underwent a multimodal approach corroborating the study of Gonzalez-Iglesias and collaborators (2011) that rehabilitated patients with lateral epicondylalgia with association techniques.

Herd and Meserve (2008), conducted a systematic review to assess the effectiveness of manipulative therapy in lateral epicondylitis, according to the authors the mobilization with movement provides short and long term benefits in treated individuals.

Paungmali and collaborators (2003), conducted a study to evaluate the effect Sympathoexcitatory and hypoalgesic the mobilization with movement on the side of epicondylalgia, according to the authors, MWM has a physiological effect similar to that seen in the manipulation of the spine. Abbott (2001), states that the MWM improves shoulder range of movement (ROM) in patients with lateral epicondylitis, in the affected side and the unaffected side, according to this author, the MWM can lead to decreased muscle tone mediated neurophysiologically. Paungmali et al. (2006) reported that the technique of mobilization with movement has rapid effect in reducing immediate pain, followed by the progression of improved function. Corroborating with these authors was observed improved function and pain of the volunteers treated this search.

Agreement with our study Pagorek (Miller 2000) reports that the benefit of this intervention in clinical practice is that it is faster compared to other methods of treatment, requiring no additional equipment and results in sudden attenuation of pain and improved function. In this study, it was observed attenuation of pain and it made possible the development of eccentric exercise associated with MWM. In the present study, we used the standardized weight 1 kgf, this load was chosen because it is a low load and all the volunteers were able to perform the task without that promotes pain after the session, in the course of weeks there was an increase in the number of repetitions of the exercise.

However, Slater and collaborators (2006) in their study showed there was no significant analgesic effects in the short term in response to mobilization with movement, the present study the improvement in pain was observed and hence improved function, since the volunteers were able to perform exercise proposed when we conduct mobilization.

The eight volunteers reported improvement in pain during the performance of mobilization with movement of the elbow. Since according to Vicenzino and collaborators (2001), the MWM provides improved pain symptoms of patients, and infers that the improvement in pain at the start of a rehabilitation program may accelerate the recovery or better motivate the patient to persist in the proposed treatment program (Vicenzino 2003).

Cullnane et al. (2014) and Peterson et al. (2014), report that eccentric exercise of extensor muscles of wrist and fingers, reduce pain and improve grip strength in chronic individuals and suggest that these exercises can be included as part of multimodal treatment of lateral epicondylitis, being that, in present study was performed at multimodal methodology with eccentric exercise associated with mobilization with movement. By contrast, Heijnders and Lin (2015) reported the need for further studies to confirm the effectiveness established by this technique and the size of the effect of eccentric exercise in lateral epicondylitis.

Fernández-Carnero and collaborators (2007, 2008), They conducted a controlled clinical study and blind, to assess the presence of active and latent trigger point in patients with lateral epicondylalgia, according to these authors triggers points are present in the forearm muscles of the patients and contribute in pain on the affected side. The current study corroborates the studies cited above it was observed presence of point triggers on volunteers, so was held inhibition of point triggers the extensor muscles of the wrist and fingers.

Our study had some limitations for the benefit of the results as absence of surface electromyography, the dynamometer device as an aid to assess the pre and post treatment and the number of volunteers. We suggest that there is a need conduct a randomized clinical study using the techniques used in this study in different groups to observe the effect of the interaction of these techniques in the rehabilitation in a greater number of the patients with lateral epicondylosis.

## Conclusion

According to the data obtained, we deduced that the multimodal rehabilitation was effective in sample evaluated, was observed a significant reduction in pain and improvement in function of individuals with lateral epicondylosis evaluated in this case series.
